# Investigating the Variables Associated with Physical Exercise Status among United States Adults with Arthritis

**DOI:** 10.3390/jcm13154526

**Published:** 2024-08-02

**Authors:** David R. Axon

**Affiliations:** Department of Pharmacy Practice & Science, R. Ken Coit College of Pharmacy, The University of Arizona, 1295 N. Martin Ave., Tucson, AZ 85721, USA; draxon@arizona.edu; Tel.: +1-520-621-9561

**Keywords:** exercise, physical activity, arthritis, associations, Medical Expenditure Panel Survey, cross-sectional, United States adults

## Abstract

**Background/Objectives:** Arthritis is a chronic, debilitating condition affecting millions of United States (US) adults. Regular physical exercise is particularly important for adults with arthritis. This study aimed to investigate the characteristics associated with regular physical exercise in US adults with arthritis. **Methods:** This cross-sectional database study used 2021 Medical Expenditure Panel Survey data and included US adults (age ≥ 18) alive with arthritis. A multivariable logistic regression model was developed to test the association of the following variables with regular physical exercise (defined as moderate-vigorous intensity exercise for ≥30 min ≥5 times weekly; yes, no): age, sex, Hispanic, race, census region, marriage status, schooling, employment, health insurance, household income, mental health, general health, smoking status, chronic conditions, pain, and functional limitations. **Results:** Overall, 5091 people (regular physical exercise *n* = 2331, no regular physical exercise *n* = 2760) were involved in this analysis. Most were female, non-Hispanic, white, married, had schooling beyond high school, were unemployed, had private health insurance, had mid-high household income, had good mental health, had good general health, were non-smokers, had two or more chronic conditions, had little/moderate pain, and did not have a functional limitation. In multivariable logistic regression analysis, male vs. female sex (odds ratio [OR] = 1.440, 95% confidence interval [CI] = 1.185–1.749), employed vs. unemployed (OR = 1.277, 95% CI = 1.005–1.624), good vs. poor general health (OR = 2.174, 95% CI = 1.673–2.824), little/moderate vs. quite a bit/extreme pain (OR = 1.418, 95% CI = 1.109–1.818), and no functional limitation (OR = 1.592, 95% CI = 1.282–1.980) were associated with higher odds of reporting regular physical exercise, while Midwest vs. West census region (OR = 0.698, 95% CI = 0.521–0.935) was associated with lower odds of reporting regular physical exercise. **Conclusions:** This study identified variables associated with regular physical exercise among US adults with arthritis. Further work is needed to develop interventions for characteristics that may help increase exercise and, subsequently, health outcomes in this population.

## 1. Introduction

Arthritis is a chronic, often debilitating, condition that involves inflammation, swelling, and pain or tenderness of joints in the body [1]. Arthritis affected approximately 53 million United States (US) adults between 2019 and 2021 [2]. The prevalence of arthritis (age-adjusted) was approximately 19% in recent years (2019–2022) [2,3]. The proportion of adults with arthritis is predicted to rise in future, with one study estimating that over 78 million US adults (approximately 26% of US adults) will have a diagnosis of arthritis by the year 2040 [4]. Arthritis can affect adults of any age, although it commonly begins in middle age and increases with age [3]. Arthritis is more common among women than men [2,3], and the prevalence of arthritis also varies among several personal characteristics, such as race/ethnicity, education, household income, urbanization, and region of the US [3].

There are over 100 types of arthritis-related diseases, though osteoarthritis (a degenerative disease characterized by deterioration of joints and related tissue) and rheumatoid arthritis (an autoimmune disorder with the potential to impact many parts of the body, including joints) are the two major types [5]. Despite their differences, both osteoarthritis and rheumatoid arthritis have commonalities in risk factors and implications of the disease [6]. Symptoms of osteoarthritis can include bone spurs, grating sensations, pain, reduced flexibility, stiffness, swelling, and tenderness [1]. Symptoms of rheumatoid arthritis can include swelling, tenderness, or warmth at the joints, joint stiffness (especially after inactivity or waking up), appetite loss, fatigue, and fever [1]. Common risk factors for arthritis include family history (increased risk if parents or siblings have arthritis), age (increased risk with aging), sex (increased risk among women), joint injury (increased risk from previous joint injuries), and obesity (increased risk with a larger weight due to extra pressure on body joints) [1]. Genetics are also associated with the development of osteoarthritis and rheumatoid arthritis [7,8]. Many genes are associated with the development of rheumatoid arthritis, most notably the human leukocyte antigen (HLA)-DRB1 gene and the protein tyrosine phosphate 22 (PTPN22) gene [9].

Arthritis is among the top causes of disability and one of the costliest diseases to manage in the US, resulting in several disabilities and functional limitations [10,11]. Arthritis can also negatively affect a person’s physical, psychological, and social aspects of quality of life and mental health [12,13]. People with painful conditions, such as arthritis, use a substantial number of medications and medical strategies that can be burdensome to manage [14]. Furthermore, arthritis has had a substantial economic impact, equivalent to over 1% of the US Gross Domestic Product (GDP) in recent years [15]. Arthritis was associated with over $300 billion in medical expenses and lost earnings in 2013, according to a MEPS analysis that used multi-stage regression modeling for arthritis-attributable medical expenditures and lost earnings [16], while other studies have demonstrated considerable expenditures for care and unaffordable out-of-pocket costs in some cases [10].

The Arthritis Workgroup, part of the Healthy People 2030 initiative, has developed four objectives for adults with arthritis: reduction in the proportion of people with (1) moderate to severe joint pain, (2) activity limitations, (3) work limitations, and (4) not being counseled about physical exercise [17].

Physical exercise is a well-known factor that is related to a person’s health status. It is recommended that US adults partake in 30 min or more of moderate-to-vigorous-intensity physical exercise on five or more days of the week as part of a healthy lifestyle [18]. The benefits of regular physical exercise include weight management, reduced risk for several diseases (including cardiovascular disease, diabetes and other metabolic syndromes, infectious diseases, and some cancers), and increased bone, joint, and muscle strength [18]. Although physical exercise is typically beneficial for all people, it is especially important for adults with arthritis. A recent analysis using 2019 behavioral risk factor surveillance system (BRFSS) data identified walking as one of the most common physical exercise activities conducted by US adults with arthritis, followed by gardening and weightlifting to a lesser extent [19].

Adults with arthritis are advised to undertake regular physical exercise to help improve or manage their condition [20]. However, research suggests that US adults with arthritis do less physical exercise than their counterparts without arthritis, with approximately one-third (36%) meeting physical exercise guidelines [21], despite the known health benefits of physical exercise in this population. However, little is known about the association of various characteristics with physical exercise, specifically among US adults with arthritis. It is therefore of interest to study the characteristics related to physical exercise in US adults with arthritis. Improving our understanding of these relationships can help develop targeted interventions to improve physical exercise among this population. Consequently, the objective of this paper was to assess the variables associated with regular physical exercise as compared to no regular physical exercise among US adults with arthritis.

## 2. Materials and Methods

This cross-sectional database study involved the Medical Expenditure Panel Survey (MEPS) 2021 data (HC-233 full-year consolidated file), which included 28,336 individuals. MEPS investigators collect data from a strategic sample of US households based on the previous year’s (2020) Centers for Disease Control and Prevention’s (CDC) National Health Interview Survey (NHIS) sampling framework. Data are collected for many MEPS interview sections using a panel design that is conducted multiple times over two years. Beyond medical expenditure data, the MEPS dataset includes a plethora of demographic and other personal characteristics, health conditions (of note for this study, these include arthritis), and other factors associated with health status (of note for this study, these include physical exercise). Together, these self-reported data constitute the MEPS household component. Two additional components, the MEPS medical provider component and the MEPS insurance component, supplement the data collected in the MEPS household component. MEPS data files are created by MEPS staff and made freely available for researchers to access via the Agency for Healthcare Research and Quality (AHRQ) website [22].

The eligibility criteria for this study were individuals alive for the entirety of 2021, aged 18 years or older, and who self-reported a diagnosis of any type of arthritis.

The dependent variable for this study was regular physical exercise as compared to no regular physical exercise. This was determined according to self-reported answers from the MEPS item that asked whether the person participated in moderate-vigorous intensity physical exercise for 30 min or more five times or more each week [23,24].

The independent variables for this study were organized using the Andersen behavioral model, which categorizes variables as predisposing, enabling, or in need [25]. Predisposing variables included age, sex, Hispanic, and race. Enabling variables included census region, marriage status, schooling, employment, health insurance, and household income. Need variables included mental health, general health, smoking status, chronic conditions, pain, and functional limitations [23,24].

Data analysis was conducted using SAS and utilized the PROC SURVEY FREQ and PROC SURVEY LOGISTIC commands (SAS Institute Inc., Cary, NC, USA). This approach allows the use of variables that maintain the structure of complex survey data, such as MEPS, by supporting the clusters and strata within the data. The analysis also used the weighting variable to obtain weighted population-based estimates of the eligible population based on the study sample. In addition, a domain analysis was conducted to distinguish the eligible population from the ineligible population while maintaining the integrity of the complex survey data structure. Together, this approach facilitates greater external validity of the findings for US adults alive for the entirety of 2021 who self-reported a diagnosis of any type of arthritis. Any differences between US adults with arthritis who reported doing regular physical exercise as compared to those who did not were assessed using a Chi^2^ test. A first multivariable logistic regression model investigated the associations between predisposing variables and regular physical exercise. A second multivariable logistic regression model investigated the associations between predisposing and enabling variables and regular physical exercise. A third multivariable logistic regression model investigated the associations between predisposing, enabling, and need variables and regular physical exercise. In all models, no regular physical exercise served as the reference group. A statistical threshold of 0.05 was selected a priori. The University of Arizona Institutional Review Board approved this study (6 June 2024; approval number: 00004719).

## 3. Results

**Figure 1:**
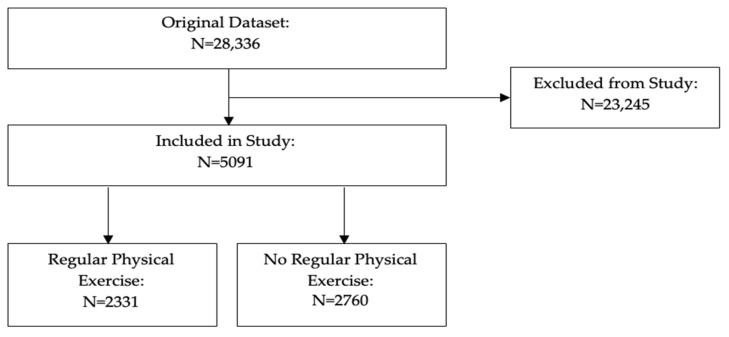
Study eligibility flowchart.

Overall, 5091 people were involved in this analysis, which represented a sample (weighted) of 63,907,189 US adults with arthritis. Among these, 2331 said they did regular physical exercise, which led to a sample (weighted) of 29,800,467 (46.6%; 95% confidence interval [CI]: 44.7%, 48.6%). Meanwhile, 2760 said they did not do regular physical exercise, which led to a sample (weighted) of 34,106,721 (53.4%; 95% CI: 51.4%, 55.3%). See Figure 1.

The most frequently reported age group was ≥70-year-olds (36.6%), with decreasing proportions of people in younger age groups. The most frequently reported census region was the South (40.2%), followed by the Midwest, West, and Northeast. Study participants were most often female (60.7%), non-Hispanic (90.9%), white (80.7%), married (53.4%), had schooling beyond high school (58.0%), were unemployed (59.1%), had private health insurance (58.2%), had mid-high household income (69.3%), had good mental health (85.5%), had good general health (74.4%), were non-smokers (86.3%), had two or more chronic conditions (64.0%), had little/moderate pain (70.8%), and did not have a functional limitation (66.1%). There were statistically significant differences between the two physical exercise groups for the following variables: sex, race, schooling, employment, household income, mental health, general health, chronic conditions, pain, and functional limitations. See Table 1.

In the final multivariable logistic regression model, male sex (vs. female), employed (vs. unemployed), good (vs. poor) general health, little/moderate pain (vs. quite a bit/extreme), and no functional limitation (vs. functional limitation) were associated with higher odds of the person reporting they did regular physical exercise. The Midwest (vs. West) census region was associated with lower odds of the person reporting they did regular physical exercise. See Table 2.

## 4. Discussion

The main findings of this paper are that male vs. female sex, employed vs. unemployed, good vs. poor general health, little/moderate vs. quite a bit/extreme pain, and no functional limitation vs. having a functional limitation were associated with higher odds of reporting regular physical exercise, while the Midwest vs. West census region was associated with lower odds of reporting regular physical exercise in multivariable logistic regression analysis. There was no association between the remaining variables and the odds of reporting regular physical exercise among US adults with arthritis. The following discussion expands upon each of these findings in turn.

Among the predisposing variables, male (vs. female) sex was associated with higher odds of the person stating they did regular physical exercise in all three models. The literature frequently describes how males do more physical exercise than females in all stages of life [26,27], with one recent report stating that 43.1% of males and 32.5% of females regularly engage in aerobic physical activity [28]. It is thought that the anatomical and physiological differences between sexes account for differences in the health outcomes of exercise [29]. Research has also demonstrated that females may benefit more from regular physical exercise than men for other conditions, such as cardiovascular mortality risk reduction [28]. Women often report a greater occurrence of arthritis, higher levels of inflammation and pain, and use more health services as compared to men, which may account for why they do less physical exercise [30]. It is therefore recommended that greater efforts be made to encourage females with arthritis to increase their time spent doing physical exercise [31].

Some of the older age categories, relative to the younger age categories, were associated with greater odds of the person stating they did regular physical exercise in model 2, but this association was not observed in the final multivariable model. Regardless, physical exercise has numerous benefits for health at all ages [32] and should therefore continue to be recommended for adults of all ages with arthritis.

There was no association between the other predisposing variables and regular physical exercise in the final model. There has been a greater occurrence of arthritis documented in White and non-Hispanic US adults [33,34], although this information may be less accurate as minorities are often underrepresented in research examining arthritis [34]. The 2006–2015 National Health Interview Survey data reported that Black, Asian, and Hispanic adults were less likely to participate in physical exercise than White or non-Hispanic adults [35]. Additional research to explore the influence of race/ethnicity on physical exercise among the US adult population with arthritis is needed.

Among the enabling variables, the Midwest (vs. West) census region was associated with lower odds of the person stating they did regular physical exercise. Differences in weather throughout the year between these large regions may influence the prevalence of arthritis, and it is possible that poor weather influenced the ability of people in the Midwest to exercise regularly outdoors during winter. It is worth considering that these four categories for the US census region may be too broad to be meaningful. More granular data or other variables, such as metropolitan status or urbanization status, may be more informative and should be considered in future work. For instance, a recent study tracking exercise activities in Georgia found that metropolitan residents did more activities and had a higher activity difficulty level than non-metropolitan residents [36].

Being employed (vs. unemployed) was correlated with increased odds of the person stating they did regular physical exercise. A possible explanation is that arthritis is more prevalent among older people [3], who are typically retired from employment. It is also possible that people suffering from extreme arthritis have left the workforce (due to pain, disability, etc.), and are therefore less likely to exercise due to their condition. There may be value in developing initiatives for unemployed adults with arthritis to participate in physical exercise appropriate to their abilities and needs.

There was no association between the other enabling variables, such as health insurance and household income, and regular physical exercise. Research has found that people with higher incomes are correlated with an increased likelihood of satisfying regular physical exercise guidelines [37]. As with the call for physical exercise initiatives for unemployed adults with arthritis, there may also be value in encouraging greater physical exercise for US adults with arthritis regardless of status for enabling variables.

Among the need variables, good general health (vs. poor) was associated with higher odds of the person stating they did regular physical exercise. This is unsurprising given the well-established association between exercise and health [38,39,40]. Likewise, it was perhaps unsurprising that those with little/moderate (vs. quite a bit/extreme) pain were associated with higher odds of the person stating they did regular physical exercise. One study reported that people with arthritis who exercised had less pain than those who did not exercise [41]. Other studies have found little/moderate pain was associated with increased odds of doing regular physical exercise compared to those with quite a bit or extreme pain, among a study of US older adults with pain [42,43]. Furthermore, no functional limitation (vs. functional limitation) was associated with greater odds of the person stating they did regular physical exercise. Physical exercise is thought to be associated with reduced functional limitations across a person’s life and in older age [44,45]. However, there was no association between mental health, smoking, and number of chronic conditions with physical exercise.

Given the increasing interest in mental health in recent years, research has identified a correlation between arthritis and mental health issues [46]. The 2015–2017 National Health Interview Survey data indicated 23% of US adults with arthritis had anxiety and 12% had depression [47]. Regular physical exercise can reduce symptoms of anxiety and depression and help improve mental health [48]. Data from the Behavioral Risk Factors Surveillance System have also reported fewer poor mental health days among those who did more physical exercise [49]. Efforts to improve mental health, through exercise or otherwise, are warranted for this population of adults with arthritis [50]. It was interesting that smoking status and the number of chronic conditions were not associated with physical exercise in this population. One might assume that being a smoker would be correlated with lower odds of doing exercise, since both are negative health attributes. Smoking is an established risk factor for arthritis; thus, smoking status is particularly important to consider in this population [5,51]. One might also assume that multiple chronic conditions would be correlated with lower odds of doing exercise, as those with more conditions may be less able to regularly participate in physical exercise. However, this was not the case in this study of US adults with arthritis. Previous research has found lower levels of physical activity in people with chronic disease as compared to those without chronic disease [52,53]. The influence of chronic conditions should be investigated further among US adults with arthritis. Overall, efforts should be made to address poor health attributes among these needed variables, alongside encouraging US adults with arthritis to do more physical exercise.

The current study also supports the findings of previous work that indicate the occurrence of regular physical exercise is too low in US adults with arthritis [31]. Overall, less than 25% of US residents meet the CDC guidelines for regular physical exercise [3]. Greater efforts, including public health policy interventions and more counseling from healthcare providers, are needed to improve physical exercise among US adults with arthritis [31,32] in accordance with physical activity recommendations [54].

Finally, to address the limited literature reporting the association of variables with physical exercise among populations of adults with arthritis specifically, more research is needed for the variables explored in this study of US adults with arthritis to further investigate and compare our findings.

Study limitations include the study design, which, as a cross-sectional database study, was only able to establish a statistical association and not a cause-and-effect relationship. In addition, the predominately self-reported nature of data from the MEPS household component may be prone to recall bias due to the time between interviews. It is possible that study subjects incorrectly reported their diagnosis of arthritis (or incorrectly reported data for any of the variables used in the study), and social desirability bias may be present from participants overestimating their physical exercise regimen. Weight, or body mass index, and genetic history are known risk factors that were not available in the dataset and thus could not be investigated in this study but could be investigated in future studies. Further research could utilize a dataset with a sufficient sample size to stratify these results by the two major types of arthritis, osteoarthritis and rheumatoid arthritis, to provide more focused insights into the association with regular physical exercise.

## 5. Conclusions

This cross-sectional database study identified characteristics statistically correlated with regular physical exercise among US adults with arthritis. Interventions are needed to target individuals with characteristics negatively associated with regular physical exercise, with the intent that they help increase the amount of physical exercise conducted among this population and optimize health outcomes for adults with arthritis. Future work would be necessary to evaluate the effectiveness of such interventions.

## Figures and Tables

**Table 1:** Characteristics of United States adults with arthritis who did regular physical exercise, no regular physical exercise, and total exercise in the weighted study population.

Variable	Regular Physical Exercise Wtd % [95% CI]	No Regular Physical Exercise Wtd % [95% CI]	Total Wtd % [95% CI]	*p*-Value
Predisposing variables				
Age				0.54
≥70 years	36.4 [33.7, 39.2]	36.8 [34.5, 39.1]	36.6 [34.8, 38.5]	
60–69 years	28.5 [26.2, 30.8]	27.7 [25.3, 30.0]	28.1 [26.4, 29.8]	
50–59 years	20.2 [17.8, 22.6]	18.8 [16.8, 20.9]	19.5 [17.9, 21.1]	
18–49 years	14.9 [12.6, 17.1]	16.7 [14.3, 19.1]	15.9 [14.2, 17.5]	
Sex				<0.001
Male	44.0 [41.3, 46.7]	35.2 [33.1, 37.3]	39.3 [37.7, 40.9]	
Female	56.0 [53.3, 58.7]	64.8 [62.7, 66.9]	60.7 [59.1, 62.3]	
Hispanic				0.21
Yes	8.4 [6.8, 10.0]	9.7 [7.6, 11.7]	9.1 [7.5, 10.6]	
No	91.6 [90.0, 93.2]	90.3 [88.3, 92.4]	90.9 [89.4, 92.5]	
Race				0.02
White	82.7 [80.3, 85.1]	79.0 [76.5, 81.4]	80.7 [78.7, 82.7]	
Black	10.9 [8.9, 12.8]	13.9 [11.9, 16.0]	12.5 [10.8, 14.2]	
American Indian/Alaska native	0.9 [0.4, 1.4]	0.5 [0.2, 0.8]	0.7 [0.4, 1.0]	
Asian/Native Hawaiian/Pacific Islander	3.3 [2.0, 4.6]	3.6 [2.4, 4.9]	3.5 [2.4, 4.5]	
Multiple races	2.2 [1.4, 2.9]	3.0 [2.1, 4.0]	2.6 [1.9, 3.3]	
Enabling variables				
Census region				0.07
Northeast	17.3 [14.1, 20.5]	17.0 [13.5, 20.5]	17.2 [14.1, 20.2]	
Midwest	21.2 [17.7, 24.7]	23.3 [19.6, 26.9]	22.3 [18.9, 25.6]	
South	39.0 [34.5, 43.6]	41.2 [36.9, 45.6]	40.2 [36.2, 44.2]	
West	22.4 [18.2, 26.7]	18.5 [15.1, 21.9]	20.3 [17.0, 23.6]	
Marriage status				0.05
Married	55.3 [52.7, 57.9]	51.8 [49.1, 54.5]	53.4 [51.4, 55.4]	
Not married	44.7 [42.1, 47.3]	48.2 [45.5, 50.9]	46.6 [44.6, 48.6]	
Schooling				<0.001
High school or less	38.2 [35.5, 41.0]	45.3 [42.8, 47.7]	42.0 [40.1, 43.9]	
More than high school	61.8 [59.0, 64.5]	54.7 [52.3, 57.2]	58.0 [56.1, 59.9]	
Employment				<0.001
Employed	46.6 [43.7, 49.5]	35.9 [33.4, 38.4]	40.9 [39.1, 42.8]	
Unemployed	53.4 [50.5, 56.3]	64.1 [61.6, 66.6]	59.1 [57.2, 60.9]	
Health insurance				0.15
Private	59.8 [57.0, 62.7]	56.9 [54.3, 59.4]	58.2 [56.2, 60.3]	
Public	37.8 [35.1, 40.4]	41.1 [38.7, 43.4]	39.5 [37.7, 41.4]	
None	2.4 [1.4, 3.4]	2.1 [1.3, 2.9]	2.2 [1.5, 3.0]	
Household income				<0.001
Low	26.5 [24.0, 29.0]	34.5 [31.8, 37.2]	30.7 [28.8, 32.7]	
Mid/high	73.5 [71.0, 76.0]	65.5 [62.8, 68.2]	69.3 [67.3, 71.2]	
Need variables				
Mental health				<0.001
Good	90.0 [88.4, 91.6]	81.6 [79.7, 83.4]	85.5 [84.3, 86.7]	
Poor	10.0 [8.4, 11.6]	18.4 [16.6, 20.3]	14.5 [13.3, 15.7]	
General health				<0.001
Good	85.7 [83.7, 87.6]	64.6 [62.1, 67.1]	74.4 [72.8, 76.0]	
Poor	14.3 [12.4, 16.3]	35.4 [32.9, 37.9]	25.6 [24.0, 27.2]	
Smoking status				0.71
Current smoker	13.4 [11.3, 15.5]	13.9 [12.2, 15.6]	13.7 [12.3, 15.1]	
Nonsmoker	86.6 [84.5, 88.7]	86.1 [84.4, 87.8]	86.3 [84.9, 87.7]	
Chronic conditions				<0.001
2 or more	58.9 [56.0, 61.7]	68.5 [66.3, 70.7]	64.0 [62.2, 65.8]	
Less than 2	41.1 [38.3, 44.0]	31.5 [29.3, 33.7]	36.0 [34.2, 37.8]	
Pain				<0.001
Little/moderate	80.8 [78.0, 83.6]	63.5 [60.5, 66.5]	70.8 [68.6, 72.9]	
Quite a bit/extreme	19.2 [16.4, 22.0]	36.5 [33.5, 39.5]	29.2 [27.1, 31.4]	
Functional limitation				<0.001
No	76.5 [74.1, 78.8]	56.9 [54.2, 59.7]	66.1 [64.2, 67.9]	
Yes	23.5 [21.2, 25.9]	43.1 [40.3, 45.8]	33.9 [32.1, 35.8]	

Wtd = weighted. CI = confidence interval. The difference between regular physical exercise and no regular physical exercise groups for each variable was assessed using the Chi^2^ test.

**Table 2:** Associations of variables with regular physical exercise (vs. no regular physical exercise) among United States adults with arthritis.

Variable	Model 1: Predisposing Variables OR [95% CI]	Model 2: Predisposing + Enabling Variables OR [95% CI]	Model 3: Predisposing + Enabling + Need Variables OR [95% CI]
Predisposing variables:			
Age: ≥70 years vs. 18–49 years	1.075 [0.827, 1.396]	**1.456 [1.092, 1.941]**	1.175 [0.808, 1.709]
Age: 60–69 years vs. 18–49 years	1.126 [0.856, 1.480]	**1.366 [1.022, 1.827]**	1.144 [0.804, 1.628]
Age: 50–59 years vs. 18–49 years	1.166 [0.873, 1.558]	1.241 [0.922, 1.669]	1.222 [0.834, 1.790]
Sex: male vs. female	**1.424 [1.223, 1.657]**	**1.403 [1.203, 1.636]**	**1.440 [1.185, 1.749]**
Hispanic: yes vs. no	0.867 [0.672, 1.119]	0.873 [0.668, 1.142]	0.848 [0.598, 1.205]
Race: White vs. multiple	1.407 [0.922, 2.148]	1.346 [0.872, 2.075]	1.059 [0.554, 2.026]
Race: Black vs. multiple	1.055 [0.687, 1.621]	1.073 [0.695, 1.658]	0.861 [0.445, 1.666]
Race: American Indian/Alaskan vs. multiple	**2.582 [1.003, 6.647]**	2.697 [0.970, 7.498]	1.974 [0.618, 6.305]
Race: Asian/Hawaiian/Pacific Islander vs. multiple	1.219 [0.651, 2.281]	1.034 [0.559, 1.914]	0.572 [0.231, 1.420]
Enabling variables:			
Census region: Northeast vs. West		0.839 [0.624, 1.126]	0.893 [0.650, 1.225]
Census region: Midwest vs. West		**0.741 [0.572, 0.961]**	**0.698 [0.521, 0.935]**
Census region: South vs. West		0.809 [0.625, 1.047]	0.915 [0.702, 1.191]
Marriage status: married vs. not married		1.009 [0.867, 1.173]	0.849 [0.699, 1.031]
Schooling: high school or less vs. more than high school		**0.817 [0.706, 0.946]**	0.976 [0.816, 1.167]
Employment: employed vs. unemployed		**1.648 [1.375, 1.974]**	**1.277 [1.005, 1.624]**
Health insurance: private vs. none		0.708 [0.427, 1.173]	0.855 [0.459, 1.592]
Health insurance: public vs. none		0.800 [0.486, 1.315]	1.007 [0.536, 1.892]
Household income: low vs. mid/high		0.844 [0.706, 1.008]	0.940 [0.753, 1.175]
Need variables:			
Mental health: good vs. poor			1.003 [0.760, 1.324]
General health: good vs. poor			**2.174 [1.673, 2.824]**
Smoking status: current smoker yes vs. nonsmoker			1.205 [0.898, 1.616]
Chronic conditions: 2 or more vs. less than 2			0.798 [0.634, 1.006]
Pain: little/moderate vs. quite a bit/extreme			**1.418 [1.109, 1.818]**
Functional limitation: no vs. yes			**1.592 [1.282, 1.980]**

OR = odds ratio. CI = confidence interval. Statistically significant results identified in bold. All Wald statistics < 0.001. C-statistics: model 1 = 0.56; model 2 = 0.59; model 3 = 0.66.

## Data Availability

No new data were created or analyzed in this study. Data sharing is not applicable to this article.
